# Reliability and Validity of an Ultrasound-Based Protocol for Measurement of Quadriceps Muscle Thickness in Children

**DOI:** 10.3389/fphys.2022.830216

**Published:** 2022-06-27

**Authors:** Emil Rydell Högelin, Kajsa Thulin, Ferdinand von Walden, Lotta Fornander, Piotr Michno, Björn Alkner

**Affiliations:** ^1^ Department of Biomedical and Clinical Sciences, Linköping University, Linköping, Sweden; ^2^ Futurum - Academy for Health and Care, Jönköping, Sweden; ^3^ Department of Orthopaedic Surgery, Eksjö, Jönköping, Sweden; ^4^ Department of Paediatrics, Karolinska University Hospital, Stockholm, Sweden; ^5^ Department of Women’s and Children’s Health, Karolinska Institutet, Stockholm, Sweden; ^6^ Department of Orthopedic Surgery, Norrköping, Sweden; ^7^ Department of Orthopaedic Surgery, Jönköping, Sweden

**Keywords:** ultrasonography, skeletal muscle, children, magnetic resonance imaging, hypertrophy, atrophy

## Abstract

**Introduction and aims:** Accurate determination of skeletal muscle size is of great importance in multiple settings including resistance exercise, aging, disease, and disuse. Ultrasound (US) measurement of muscle thickness (MT) is a method of relatively high availability and low cost. The present study aims to evaluate a multisite ultrasonographic protocol for measurement of MT with respect to reproducibility and correlation to gold-standard measurements of muscle volume (MV) with magnetic resonance imaging (MRI) in children.

**Material and methods:** 15 children completed the study (11 ± 1 year, 41 ± 8 kg, 137 ± 35 cm). Following 20 min supine rest, two investigators performed US MT measurements of all four heads of the *m. quadriceps femoris*, at pre-determined sites. Subsequently, MRI scanning was performed and MV was estimated by manual contouring of individual muscle heads.

**Results:** Ultrasound measurement of MT had an intra-rater reliability of ICC = 0.985–0.998 (CI 95% = 0.972–0.998) and inter-rater reliability of ICC = 0.868–0.964 (CI 95% = 0.637–0.983). The US examinations took less than 15 min, per investigator. Muscle thickness of all individual quadriceps muscles correlated significantly with their corresponding MV as measured by MRI (overall *r =* 0.789, *p* < 0.001)*.*

**Conclusion:** The results of this study indicate that US measurement of MT using a multisite protocol is a competitive alternative to MRI scanning, especially with respect to availability and time consumption. Therefore, US MT could allow for wider clinical and scientific implementation.

## 1 Introduction

An accurate determination of skeletal muscle mass is of great importance when investigating muscle adaptation during hypertrophy (Aagaard et al., 2001; Morse et al., 2005; D'Antona et al., 2006; Franchi et al., 2018) and atrophy (Abe et al., 1997; LeBlanc et al., 2000; Alkner and Tesch, 2004; Maurits et al., 2004; Rittweger et al., 2005; Petterson et al., 2008; Dirks et al., 2016; Johnson et al., 2018). Several methods for assessing or estimating skeletal muscle size have been described including bioimpedance (Salinari et al., 2002), computerized tomography (Van Roie et al., 2013), magnetic resonance imaging (MRI) (Walton et al., 1997; Alkner and Tesch, 2004; Nordez et al., 2009) and ultrasound (US) (Miyatani et al., 2002; Miyatani et al., 2004; Reeves et al., 2004; Sanada et al., 2006; Moreau et al., 2010; Baldwin et al., 2011; Scott et al., 2012; Strasser et al., 2013; Tillquist et al., 2014; Fukumoto et al., 2015; Giles et al., 2015; Nakatani et al., 2016; Hadda et al., 2017; Matta et al., 2017; Stock et al., 2017; Franchi et al., 2018; Pardo et al., 2018; Filippo et al., 2019; Mechelli et al., 2019; Cheon et al., 2020; Betz et al., 2021; Lee et al., 2021; Mpampoulis et al., 2021; Takahashi et al., 2021). Muscle volume (MV) estimated from multiple cross-sectional areas (CSA) using MRI is considered the current gold standard, however, this process is time consuming and labor-intensive (Nordez et al., 2009), therefore the feasibility of MRI is limited, especially for repeated measurements in large cohorts. Alternatives like computerized tomography could also be utilized, however, the exposure to ionizing radiation makes it unsuitable, especially for children (Nakayama et al., 2019). On the contrary, ultrasonography (US) is a method that allows quick and mobile scanning at a low cost without ionizing radiation.

For a protocol of US scanning to be useful, it needs to be reliable throughout repeated scans (i.e., intra and inter-rater reliability) (Hadda et al., 2017; Pardo et al., 2018; Filippo et al., 2019; Takahashi et al., 2021) and be valid to reference modalities (i.e., MRI-estimated MV) (Miyatani et al., 2002; Miyatani et al., 2004; Franchi et al., 2018; Liegnell et al., 2021). However, the literature is mainly focused on measuring the Muscle thickness (MT) of *m. rectus femoris* (RF) and to some extent, the thickness of *m. vastus intermedius* (VI) underneath (Moreau et al., 2010; Galindo Martín et al., 2017; Hadda et al., 2017; Pardo et al., 2018; Takahashi et al., 2021). Some have investigated the other muscles of the *m. quadriceps femoris* (QF) (Moreau et al., 2010; Franchi et al., 2018; Cheon et al., 2020; Betz et al., 2021; Mpampoulis et al., 2021), and others have utilized a multisite approach, measuring all the individual muscles of the QF (Cadore et al., 2012; Strasser et al., 2013; Matta et al., 2017; Santos and Armada-da-Silva, 2017; Lee et al., 2021). These studies utilized varying methodologies, and none have provided conclusive evidence of both validity, compared to MRI, and intra and inter-rater reliability. Consequently, data on both reliability and validity of an easy-to-use US scanning protocol encompassing all heads of the QF is lacking, especially in children. Children, especially pre-pubertal, could have differing muscle distribution and properties compared to adults, however, data regarding this is to our knowledge lacking.

We aimed to establish a time-effective ultrasound method for determining MT of all four heads of the QF, that is, applicable to children and correlates to MRI-measured MV. We hypothesized that such a protocol could be reliably performed by two independent investigators and that the results would have a strong correlation to MRI-measured muscle volume.

## 2 Material and Methods

### 2.1 Participants

Fifteen healthy children (7 females and 8 males) with mean age, body mass, and height ±SD of 11 ± 1 year, 41 ± 8 kg and 137 ± 35 cm, were included. Recruitment was carried out at a local primary school with children aged 10–12 years in Eksjö, Sweden. The children volunteered to participate in a pilot study focusing on resistance exercise in children, including bilateral US and MRI examinations of the thigh. Informed assent was obtained from the children, after receiving age-appropriate information. The legal guardians provided both written and verbal informed consent. The study was approved by the Swedish Ethical Review Authority (EPM DNR: 2020-07112) and was conducted in accordance with the Declaration of Helsinki.

### 2.2 General Design

The participants underwent US followed by MRI on the same occasion. All participants were instructed to refrain from heavy sports, exercise, and physical education 24 h prior to MRI and US scanning. Prior to the US and MRI all participants remained in supine rest for a minimum of 20 min to avoid differences in muscle size due to skeletal muscle fluid shifts caused by gravitational forces (Berg et al., 1993). The participants were transported in the supine position from US to the MRI facility.

### 2.3 Ultrasound

A b-mode ultrasound machine (FlexFocus 500, BK Medical, Herlev, Denmark) was used with fixed settings (Db = 75, mHz = 15, Hz = 3/26) for all ultrasound examinations, only the depth was altered. A linear probe (Linear Array 8670, BK Medical) was used with the ultrasound machine. Images were stored directly on the machine. During scanning, participants remained relaxed in a supine position, feet fixed in a u-shaped pillow (Lassekudde, Solann, Stockholm, Sweden). Great care was taken to eliminate tissue compression by the probe, ensured using ample amounts of water-soluble transmission gel and the preservation of an intact curvature of the skin. The ultrasonographic investigation was performed by two investigators with limited previous sonographic experience. However, they underwent training for approximately 3 h prior to this study by an experienced sonographer.

The probe was placed in an axial orientation, perpendicular to the quadriceps femoris muscle. The points of measurement were performed at set intervals along the axis derived from measuring the distance from spina iliaca anterior superior (SIAS) to the proximal edge of patella.

**FIGURE 1:**
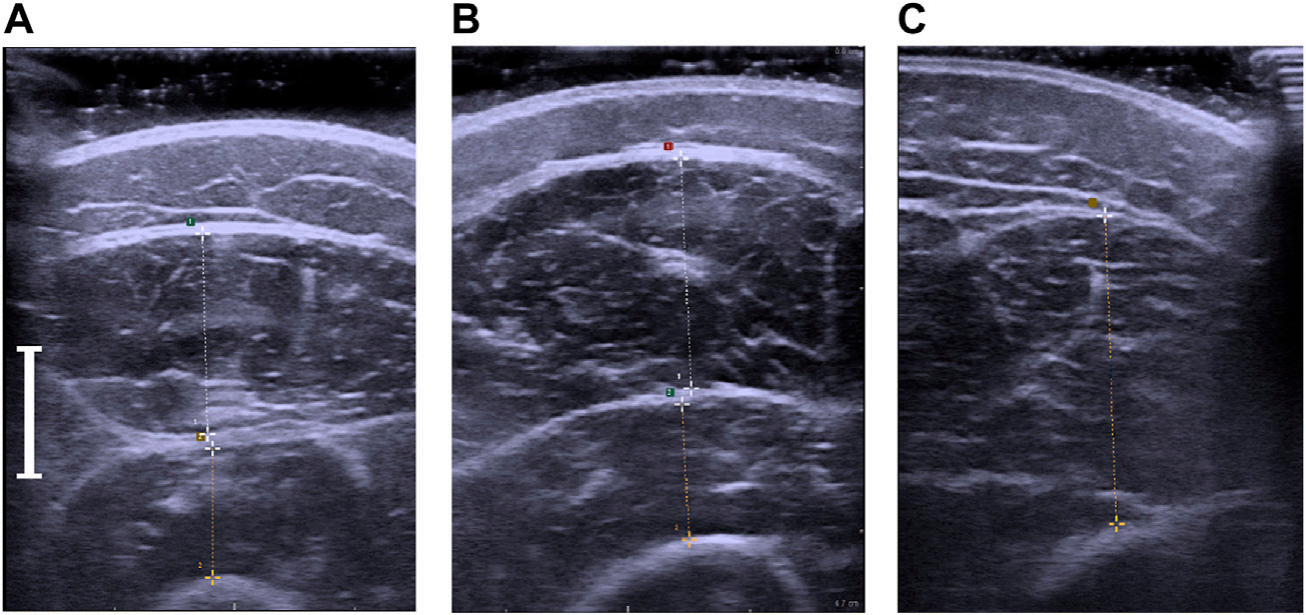
**(A)**. Ultrasonographic measurement of the m. rectus femoris and m. vastus intermedius. **(B)** Ultrasonographic measurement of the m. vastus lateralis and m. vastus intermedius. **(C)** Ultrasonographic measurement of the m. vastus medialis. The white indicator shows the length of 1 cm.

During initial pre-study trials, the inter-rater reliability of SIAS to patella measurement was deemed to have sufficient reproducibility. Furthermore, in our planned future implementation we intend to utilize a guiding ink-marking in-between measurements. Therefore, the same measurement of SIAS to patella was used. However, each of the investigators performed independent measurement of the distance. Both legs were scanned during the same session. Muscles measured were *m. rectus femoris* (*RF*), *m. vastus lateralis* (*VL*), *m. vastus intermedius* (*VI*), and *m. vastus medialis* (*VM*), exact measurement sites are shown in Table 1 *.* The point of measurement for RF (Giles et al., 2015; Stock et al., 2017; Pardo et al., 2018; Cheon et al., 2020; Takahashi et al., 2021), VI (Giles et al., 2015; Cheon et al., 2020), VL (Giles et al., 2015) and VM (Giles et al., 2015) were derived from previous literature and experience from pretesting. At each of the measuring sites three pictures were taken and evaluated. If the variance of MT exceeded 10 percent, measured from the lowest value to the highest one, additional measurements were taken. Measurements varying more than 10 percent of the mean were excluded. This process was repeated until three values not exceeding the 10 percent threshold were acquired. Measurements of MT were performed directly during the scanning in machine native software, Figure 1.

**TABLE 1:** Sites of measurement and muscles measured at each site.

Muscle	Site of measurement
RF	½ between anterior superior iliac spine and the proximal aspect of the patella, at the point of greatest thickness
VL	½ between anterior superior iliac spine and the proximal aspect of the patella, at the point of greatest thickness
VI	Measured at the same site as both RF and VL, directly underneath the measured muscle thickness of RF and VL. Mean MT of both measurements reported as VI.
VM	¾ between anterior superior iliac spine and the proximal aspect of the patella. The medial portion of the patella linear to the lateral aspect of the probe

Depicting the exact points of ultrasound measurement, RF = *m. rectus femoris*, VL = m*. vastus lateralis*, VI = m. *vastus intermedius* and VM = m. *vastus medialis*.

### 2.4 Magnetic Resonance Imaging

All the participating children tolerated the MRI scan without any discomfort. During scanning, participants were placed in a similar position to that used during ultrasound scans. An initial scout image was taken to identify the area of interest, from SIAS to tibiofemoral articulating surfaces of the knee joint. The scans were performed using a 1.5 T MRI scanner (Siemens Symphony Tim, Munich, Germany) to acquire 52 slices of axial images. One T1-weighted scan was performed using the following settings: Field of view 400 mm, repetition time = 637 ms, echo time = 12 ms, voxel size = 1.7 × 1.3 × 7.0 mm, slice thickness = 7.0 mm and interslice thickness = 1.4 mm.

#### 2.4.1 Postprocessing

**FIGURE 2:**
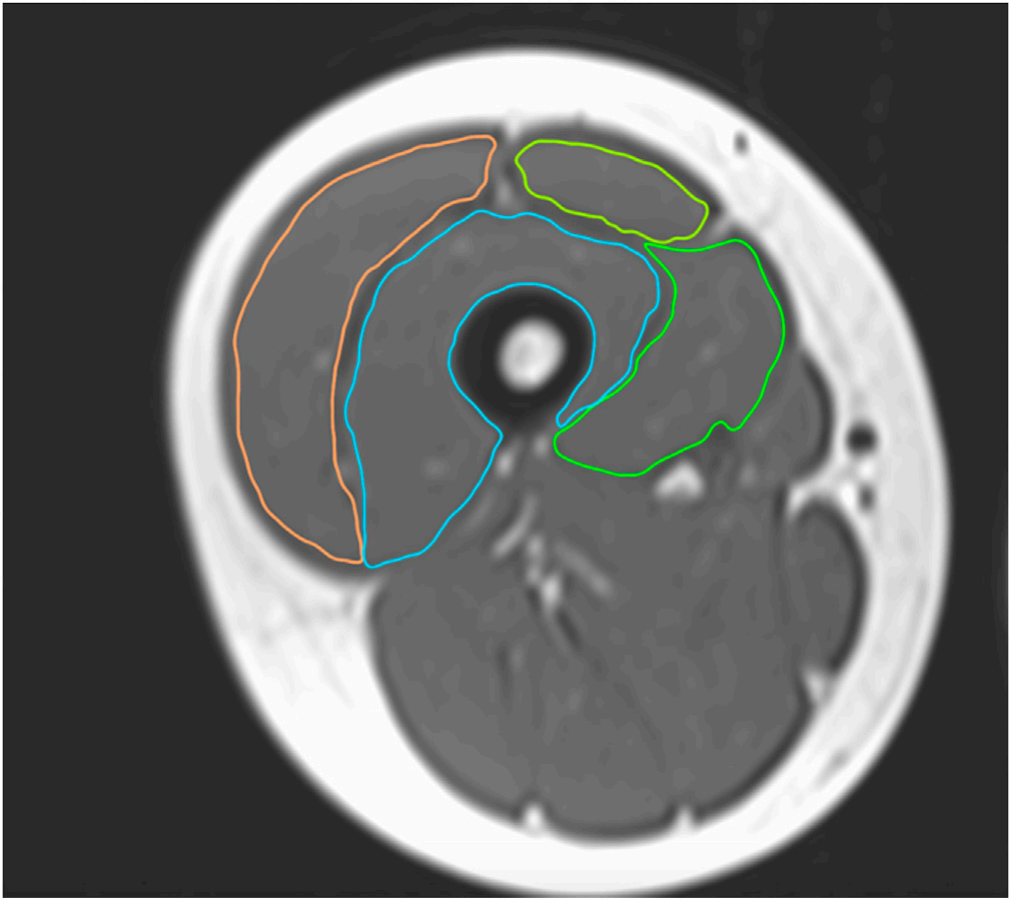
The contouring process of the m. quadriceps femoris for MV estimation of the individual muscle heads.

Image processing was performed in Osirix lite (Pixmeo, Geneva, Switzerland) for manual identification of the quadriceps muscle and contouring. Contouring was performed using a drawing tablet and pen (Wacom Intuos M, Wacom, Saitama, Japan). Contours of RF, VM, VI, and VL were drawn individually, Figure 2. We aimed to contour only muscle tissue and care was taken to exclude the fascia. No consideration for intramuscular non-contractile tissue (e.g., fat) was taken. Every second MRI slice was contoured. Determination of MV was performed using Cavalieri’s approximation (Walton et al., 1997; Nordez et al., 2009), Eq. (1).

$$\mathrm{Muscle volume}=\underset{n}{\sum }e_{i}\;\times \mathrm{CS}A_{i}$$ (1)

Equation 1: Where n is the available slices, CSA_i_ is the cross-sectional area at slice i and e_i_ is the distance between i and i+1.

A total of 623 unilateral MRI slices were contoured at a pace of approximately 40 slices per hour. Most slices included all four heads of the QF. However, for the most proximal portion of the thigh, mainly RF was detected and in the distal aspect of the thigh, only VM was delineated.

### 2.5 Intra- and Inter-Rater Reproducibility of Ultrasound Scanning and Magnetic Resonance Imaging Postprocessing

Two investigators performed ultrasound scans on all the participants in a standardized fashion as previously described. Intra-rater reproducibility was calculated from the three separate scans performed in the same session. Inter-rater reproducibility was calculated from the mean of both investigators’ measurement of each muscle.

The postprocessing, i.e., contouring of MRI images, was performed by one investigator. Intra-rater reliability was calculated from repeated contouring of 10 percent of total slices (*n* = 63), randomly selected. Both measurements were performed by the same contourer but separated in time by over a month.

### 2.6 Statistics

The statistical analyses were performed using SPSS statistics 27 (IBM, Armonk, NY, 2020). Initially, descriptive statistics were used to characterize the dataset. Thereafter, intraclass correlation coefficient (ICC) was calculated to identify reproducibility between repeated measurements, between sonographers. A two-way mixed model, single measures and absolute agreement were used for the evaluation of inter-rater reproducibility. A two-way mixed model, average measures and absolute agreement was used to evaluate intra-rater reproducibility. Intraclass correlation coefficient values were interpreted by the ranges defined by Koo et al. (Koo and Li, 2016). Standard error of measurement (SEM) and minimal detectable change (MDC) was calculated from standard deviation and ICC as described by Filippo et al. (Filippo et al., 2019). Initial descriptive statistics showed that multiple variables had a non-normally distributed pattern. Therefore, Spearman correlation was used to allow for comparisons between non-normally distributed and normally distributed variables. Values of *p* < 0.05 were considered significant.

## 3 Results

### 3.1 Ultrasonography

**FIGURE 3:**
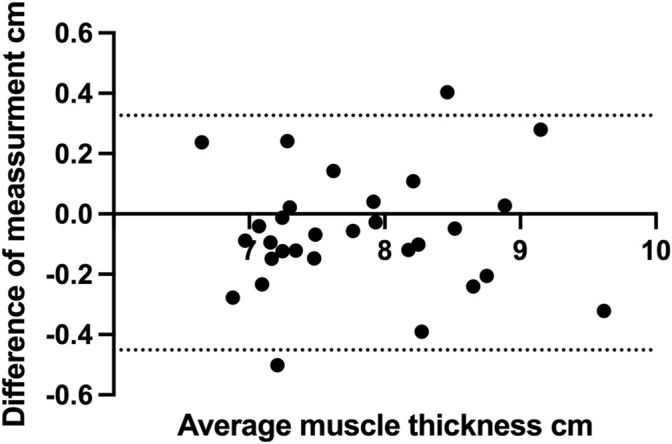
Bland-Altman graph depicting the difference between the two investigators in respect to total muscle thickness measured by ultrasound.

Ultrasonographic scanning and measurements of both thighs took less than 15 min per investigator and participant. Out of a total of 450 measurements (three measurement sites, five measurements in total, three repetitions, two limbs and 15 participants), 20 measurements exceeded >10% variance in range and were hence excluded, this accounted for 4.4% of the total measurements. Intraclass correlations between the two investigators measurements of the SIAS-patella distance was ICC = 0.936 (CI 95% 0.860–0.966). Mean values of measured MT per muscle and investigator are shown in Table 2, complemented by the mean range of the repeated measurements and the intra- and inter-rater reliability measured by ICC. The agreement of the two investigators regarding total MT is depicted in Figure 3.

**TABLE 2:** Intra- and inter-rater reliability of US scanning.

	Mean (mm)	Mean range (mm)	ICC (CI 95%) (data without exclusion)	SEM (mm)	MDC (mm)	Mean (mm)	Mean range (mm)	ICC (CI 95%) (data without exclusion)	SEM (mm)	MDC (mm)	ICC (CI 95%) (data without exclusion)	SEM (mm)	MDC (mm)
RF	18.76	0.58	0.995 (0.991–0.997) [0.992 (0.986–0.996)]	0.20	0.55	18.71	0.63	0.993 (0.988–0.997) [0.992 (0.985–0.996)]	0.24	0.66	0.968 (0.933–0.984) [0.971 (0.940–0.986)]	0.5	1.39
VL	20.13	0.54	0.995 (0.990–0.997) [0.988 (0.979–0.994)]	0.18	0.49	20.15	0.69	0.990 (0.982–0.995) [0.989 (0.979–0.994)]	0.25	0.69	0.906 (0.813–0.954) [0.903 (0.807–0.953)]	0.75	2.08
VI	13.87	0.49	0.994 (0.988–0.997) [0.987 (0.975–0.993)]	0.13	0.36	13.99	0.71	0.983 (0.968–0.991) [0.987 (0.975–0.993)]	0.24	0.66	0.944 (0.888–0.973) [0.941 (0.881–0.971)]	0.42	1.16
VM	24.83	0.56	0.995 (0.992–0.998) [0.992 (0.986–0.996)]	0.20	0.55	25.37	0.70	0.991 (0.982–0.996) [0.991 (0.982–0.996)]	0.24	0.66	0.926 (0.802–0.969) [0.925 (0.813–0.967)]	0.73	2.02
Total MT	91.48	1.37	0.997 (0.994–0.998) [0.995 (0.990–0.997)]	0.42	1.16	92.22	1.57	0.996 (0.993–0.998) [0.996 (0.993–0.998)]	0.46	1.27	0.963 (0.923–0.982) [0.963 (0.918–0.983)]	1.44	3.99

Depicting the mean and mean range of muscle thickness, separated by muscle and investigator. Mean range referrers to the mean interval form lowest to highest measurement. Intraclass correlation coefficient of each triplet of measurement, between investigators one and two, separated into each of the measured muscles and a total. Furthermore, inter-rater reliability is depicted divided by muscle. Within the brackets ICC from the first three measurement including the values otherwise excluded and remeasured is shown.

RF = *m. rectus femoris*, VL = m*. vastus* lateralis, VI = m. *vastus intermedius* and VM = m. *vastus medialis*.

ICC, intra class correlation; SEM, standard error of the measurement; MDC, minimal detectable change.

### 3.2 Magnetic Resonance Imaging

**FIGURE 4:**
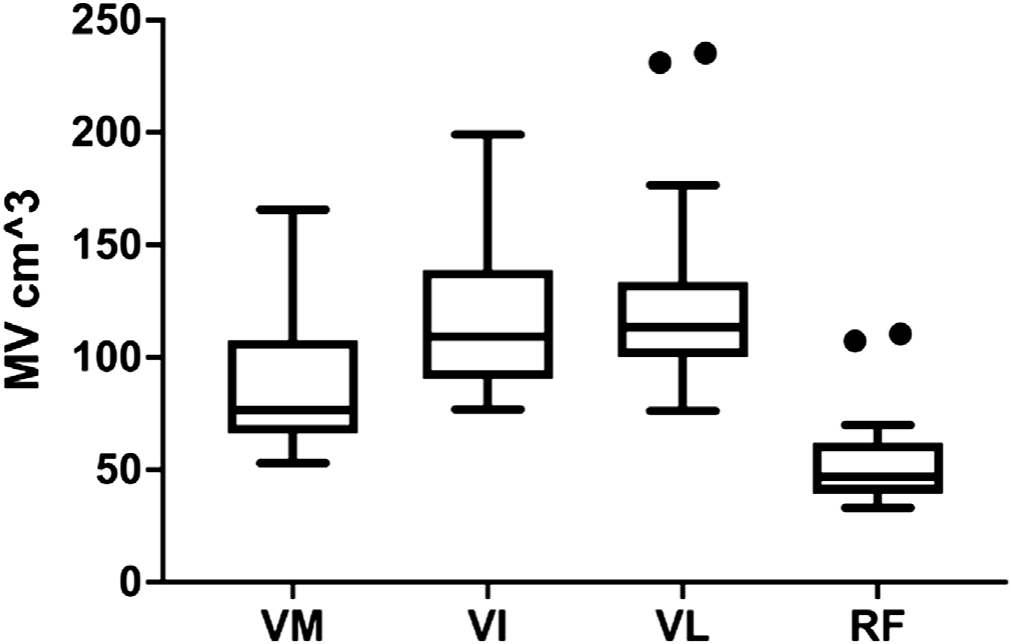
The muscle volume of VM, VI, VL and RF. The horizontal line shows the median, while the box encompasses the 50th percentile and the whiskers encompass the minimum and maximum values, outliers are displayed as points. Outliers are defined according to Tukey, as values exceeding more than the 75 percentile plus 1.5 IQR. RF = m. rectus femoris, VL = m. vastus lateralis, VI = m. vastus intermedius and VM = m. vastus medialis.

**FIGURE 5:**
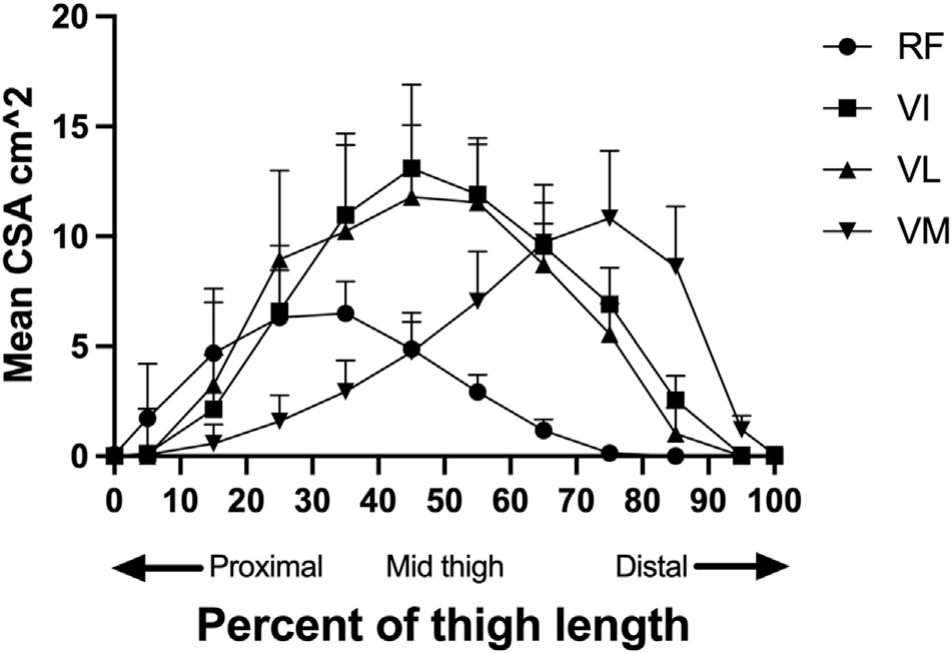
The distribution of mean muscle area of each group (RF, VI, VL, VM) along the length of the thigh, standardized for length. The standard deviation is depicted by the error bars. The first percentile is the most proximal slice where RF was still identifiable, and the one hundred percentile is the most distal slice where m. Vastus Medialis is still identifiable. RF = m. rectus femoris, VL = m. vastus lateralis, VI = m. vastus intermedius, VM = m. vastus medialis and CSA= Cross-sectional area.

The mean MV calculated by Cavalieri’s approximation is depicted in Figure 4. The VI and VL had the largest total volume closely followed by the VM, while RF had a smaller volume. The muscles also had differing dominant portions along the length of the thigh, shown in Figure 5. Repeated contouring of CSA had an ICC of 0.983 compared to initial contouring, indicative of a high degree of agreement.

### 3.3 Inter-Modality Correlation

**FIGURE 6:**
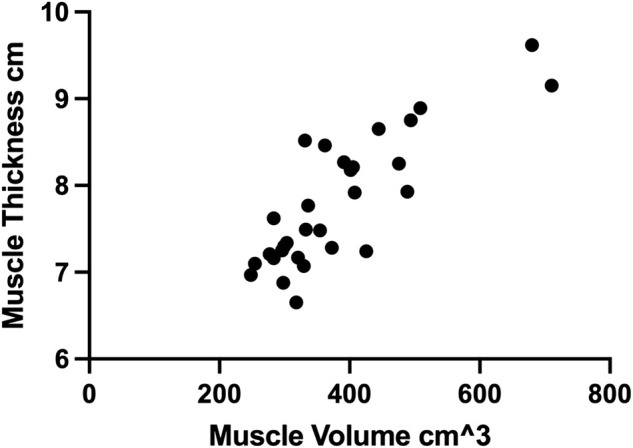
The correlation between total muscle thickness of each limb measured by ultrasound and the corresponding muscle volume measured by MRI.

Ultrasonographic measurements of MT had a correlation ranging from r = 0.514 to 0.789 with respect to their corresponding MV measured by MRI, Table 3. The total MRI MV to total US MT had the strongest correlation, shown in Figure 6. The same analysis was also performed using only the first US measurement of MT, including outliers, to evaluate the value of repeated scanning during the session, Table 3.

**TABLE 3:** Correlation between MV and MT.

	All measurement	First measurement
RF	0.702**	0.655**
VL	0.638**	0.660**
VI	0.734**	0.630**
VM	0.693**	0.724**
Total MT	0.789**	0.755**

* = *p* ≤ 0.05 ** = *p*< 0.001.

Spearman correlation coefficients between MRI measured MV and the mean US measured MT. Furthermore, correlation analysis was preformed between MRI measured MV and first value of US measured MT. All of the initial measurements of MT were used, including those who later were excluded du to variance, in all of the other analysis.

RF = *m. rectus femoris*, VL = m*. vastus lateralis*, VI = m. *vastus intermedius* and VM = m. *vastus medialis*, MT = *muscle thickness*, MV = *muscle volume*.

## 4 Discussion

The present study shows that this multisite protocol of ultrasonographic assessment of quadriceps MT can be reproduced in a reliable manner in children, supporting results from previous studies in adults (Miyatani et al., 2004; Sanada et al., 2006; Franchi et al., 2018). Our approach proved feasible to perform within a limited time frame and was easy to conduct for investigators with limited prior ultrasonographic experience. This multisite ultrasonographic protocol had a high intra- and inter-rater reliability and a strong correlation to MV measured with MRI.

Intra-rater reliability for the US measure of MT was within the “good” and “excellent” range (Koo and Li, 2016). The ICC values varied for the different muscles; however, the confidence intervals were overlapping. The current study presents a good to excellent inter-rater reliability, in line to those of previous studies (Tillquist et al., 2014; Zaidman et al., 2014; Hadda et al., 2017; Filippo et al., 2019; Mechelli et al., 2019; Betz et al., 2021; Takahashi et al., 2021). However, variations in methodology including site(s), time in-between measurements and number of measurements make direct comparisons difficult.

The ultrasonographically measured MT correlated strongly to the corresponding MRI-measured MV, similar to those of previous studies (Miyatani et al., 2002; Miyatani et al., 2004; Nakatani et al., 2016; Franchi et al., 2018). However, none of these studies employed a multisite measuring approach, thus not providing a sufficient framework of whole QF measurements to directly compare and evaluate this protocol with. Consequently, this study provides new unique results regarding the correlation of a multisite MT protocol of the *m. quadriceps femoris* to its MV.

The difference using only the first US measurement of MT instead of the mean of three after exclusion of outliers proved to be less substantial, the number of measurements could potentially be reduced and still maintain a sufficient correlation to MRI MV. A similar trend can be seen using only the first three measurements without exclusion of outliers for the reliability tests, Table 2, indicating that exclusion of outliers also had a less substantial effect.

Interestingly, *m. rectus femoris* had the smallest range of MV, Figure 3, indicating that it might constitute fundamentally different properties than the other vasti muscles. Previous studies have also shown that the RF has unique properties, as a two-joint muscle, regarding hypertrophy and activation compared to the other vasti muscles (Ema et al., 2013; Earp et al., 2015; Mangine et al., 2018). The present literature has primarily focused on RF MT and thus further studies investigating the other quadriceps are warranted.

A large aspect of the applicability of this protocol is to follow changes to specific stimuli over time, such as resistance exercise-induced hypertrophy. In this specific scenario, the value of estimating MRI measured MV is of less interest than capturing the change over time. Therefore, studies investigating repeated multisite US-measured MT over time, during the course of muscle adaptation and comparing it to MRI-measured CSA and MV, would be of great value.

## 5 Limitations

Intra-rater testing was performed in the same session, while care was taken to remove the probe from the thigh in-between measurements, a potential risk of bias remains. The use of the same measuring point between SIAS and patella, resulted in a potential bias since the investigators were not fully independent from each other’s measurements. The population investigated in this study was limited to healthy children aged 10–12 years, however, we believe this protocol would be applicable in an adult as well populations suffering from myopathology, although, this should be further confirmed.

## 6 Conclusion

This study presents a multisite ultrasound protocol for determination of quadriceps muscle thickness that has high inter-rater reliability and a strong correlation to MRI-measured MV*.* This method represents a possible alternative to MRI in settings where MRI scanning is not feasible due to time and/or financial constraints. In the case of limited resources and demand for longitudinal follow-up, multisite ultrasonographic MT measurements may represent a solid alternative.

## Data Availability

The raw data supporting the conclusions of this article will be made available by the authors, without undue reservation.
